# The Nexus of Emotional Intelligence, Empathy, and Moral Sensitivity: Enhancing Ethical Nursing Practices in Clinical Settings

**DOI:** 10.1155/jonm/9571408

**Published:** 2025-04-11

**Authors:** Esaam Khalid Ibrahim, Radhwan Hussein Ibrahim

**Affiliations:** ^1^Clinical Nursing Sciences Department, College of Nursing, University of Mosul, Mosul, Ninawa Province, Iraq; ^2^Clinical Nursing Sciences Department, College of Nursing, Ninevah University, Mosul, Ninawa Province, Iraq

**Keywords:** clinical competence, emotional intelligence, empathy, ethical decision-making, morals, nurses, patient care, professional ethics

## Abstract

**Background:** Moral sensitivity is crucial for ethical decision-making in nursing, enabling nurses to respond appropriately to ethical dilemmas in clinical settings. Emotional intelligence and empathy have been identified as key factors influencing moral sensitivity. However, limited research examines the interplay between these variables, particularly in nursing practice.

**Objective:** This study explores the relationships between emotional intelligence, nursing empathy, and moral sensitivity among nurses in Mosul teaching hospitals and examines empathy's mediating role in these relationships.

**Methods:** This study employed a descriptive cross-sectional design. It involved 300 nurses from Mosul teaching hospitals in Iraq. Data collection occurred from July 1, 2024, to October 1, 2024. Validated measurement tools were used, including the Emotional Intelligence Scale (EIS), the Jefferson Scale of Empathy, and the Moral Sensitivity Questionnaire. Statistical analyses, including Pearson's correlation, multiple regression, and mediation analysis, were conducted to examine the relationships among emotional intelligence, empathy, and moral sensitivity.

**Results:** Emotional intelligence and nursing empathy were significantly positively correlated with moral sensitivity (*r* = 0.58 and *r* = 0.66, respectively; *p* < 0.01). Multiple regression analysis revealed that both emotional intelligence (*β* = 0.30, *p* < 0.01) and nursing empathy (*β* = 0.52, *p* < 0.01) significantly predicted moral sensitivity, explaining 39% of the variance. Mediation analysis indicated that empathy partially mediated the relationship between emotional intelligence and moral sensitivity (*p* < 0.01).

**Conclusion:** This study highlights the significant roles of emotional intelligence and empathy in enhancing nurses' moral sensitivity, with empathy acting as a partial mediator. Interventions that promote emotional intelligence and empathy development in nursing education and clinical practice could improve ethical decision-making and patient care quality. Further research is needed to explore these relationships longitudinally and in diverse healthcare settings.

## 1. Introduction

The “Healthy Iraq 2030 Plan” emphasizes the need to enhance humanistic care within medical services and improve nurses' capacity to provide such care [1, 2]. Caring behavior involves nurses' deliberate actions to address patients' physical needs and emotional concerns, fostering security [3]. A lack of caring behavior among nurses can lead to increased nurse–patient conflicts and contribute to burnout [4]. Currently, nursing curricula in Iraq primarily focus on technical skills, with limited emphasis on humanistic care. Most courses are theory-based, with insufficient collaboration between nursing schools and hospitals, resulting in the neglect of humanistic care skills in practical education. This has led to nursing interns in Iraq having a low capacity for humanistic care. As these interns represent the future of the nursing workforce, their caring behavior will directly impact the quality of future clinical care [5, 6].

Moral sensitivity, also called ethical sensitivity, involves adhering to moral principles in conflict situations and being self-aware of one's roles and responsibilities [7]. It is fundamental for individuals to engage in ethical decision-making and actions. In Iraq, nurses frequently face various ethical challenges in their clinical practice, and inadequate handling of these issues can lead to increased psychological stress or even cause nurses to leave the profession. Currently, the overall moral sensitivity of nursing interns is moderate, leaving room for improvement. Research by Ye et al. indicates that the higher the moral sensitivity among nurses, the more proactive they become in delivering patient care and support [8]. Enhancing moral sensitivity in nursing staff can improve the quality of care and help stabilize the nursing workforce. Therefore, fostering a high moral sensitivity in nursing interns is crucial for improving patient care and advancing the overall development of nursing and public health [9, 10].

Emotional intelligence (EI) is the ability to perceive, understand, regulate, and manage emotions in oneself and others. According to Goleman (1995), EI comprises five key dimensions: self-awareness, which involves recognizing and understanding one's feelings; self-regulation, the ability to control emotional reactions and adapt to changing circumstances; motivation, which drives goal-oriented behavior through emotional engagement; empathy, the capacity to recognize and understand others' emotions; and social skills, which facilitate effective communication and positive interpersonal relationships. EI is crucial in enhancing communication, managing stress, and making ethical decisions in nursing. Nurses with high EI can build strong patient relationships, navigate workplace challenges, and demonstrate resilience in high-pressure environments, ultimately improving patient care and professional performance.

Empathy is the ability to understand, share, and respond to the feelings and experiences of others, making it a fundamental aspect of patient-centered care and ethical nursing practice. According to Hoffman (2000), empathy consists of cognitive empathy, which allows individuals to understand another person's perspective intellectually, and affective empathy, which enables them to resonate with others' feelings emotionally. The Jefferson Scale of Empathy highlights its importance in healthcare, emphasizing its role in improving patient outcomes, fostering trust, and enhancing professional relationships. Nurses with high levels of empathy are better equipped to provide compassionate care, recognize patients' emotional and psychological needs, and make morally sensitive decisions in clinical settings.

Despite recognizing the importance of moral sensitivity and humanistic care in nursing, there is a limited understanding of how these factors are developed and sustained among nursing interns, particularly in Iraq. Most existing studies focus on technical skill development, with minimal attention to integrating ethical sensitivity and humanistic care within the nursing curriculum. Furthermore, there is a lack of research exploring the impact of insufficient cooperation between nursing schools and hospitals on the practical training of humanistic care skills. While studies [11–13] from other countries have shown a positive relationship between high moral sensitivity and the quality of patient care, research explicitly examining this connection within the context of Iraq's healthcare system remains scarce. This research gap highlights the need for studies investigating the moral sensitivity of nursing interns in Iraq, its influence on patient care quality, and the effectiveness of integrating humanistic care into practical training programs.

This study explores the interrelationship between EI, nursing empathy, and moral sensitivity in clinical settings. Specifically, it investigates how these factors influence nurses' decision-making, patient care quality, and overall professional behavior by examining this nexus.

## 2. Theoretical Framework

This study is grounded in a multidimensional theoretical framework that integrates key constructs from established EI, empathy, and moral sensitivity theories. These components are essential for understanding the complex interplay between nurses' cognitive and affective processes in clinical decision-making and patient care.

### 2.1. Goleman's EI Theory (1995)

Goleman's framework emphasizes five core dimensions of EI: self-awareness, self-regulation, motivation, empathy, and social skills. In nursing, EI plays a crucial role in managing stress, making ethical decisions, and fostering positive relationships with patients and colleagues. This theory provides a foundation for exploring how nurses' ability to recognize and regulate emotions influences their moral sensitivity and empathy in clinical settings [14].

### 2.2. Hoffman's Theory of Empathy (2000)

Hoffman's theory of empathy explains how individuals develop empathetic responses based on emotional and cognitive processes. The theory suggests that empathy is a key driver of prosocial behavior and moral decision-making. Higher levels of empathy in nursing contribute to improved patient outcomes, compassionate care, and ethical practice. This theory supports the study by highlighting how empathy mediates the relationship between EI and moral sensitivity [15].

### 2.3. Rest's Four-Component Model of Moral Behavior (1986)

Rest's model outlines four key components necessary for moral behavior as follows:

1. Moral sensitivity: recognizing an ethical issue in a given situation.
2. Moral judgment: determining the right course of action based on ethical principles.
3. Moral motivation: prioritizing ethical values over personal interests.
4. Moral character: the perseverance to follow through with ethical actions.

Moral sensitivity is critical in the nursing profession for identifying ethical dilemmas, particularly in high-stakes patient care situations. This model provides a structured way to examine how EI and empathy contribute to nurses' ethical decision-making and professional integrity [16].

### 2.4. Integration of Theories

Combining these theoretical perspectives, this study explores the interrelationships among EI, empathy, and moral sensitivity in nursing practice. EI enables nurses to manage their emotions effectively, empathy allows them to understand and respond to patient's needs, and moral sensitivity guides them in ethical clinical decisions. This integrated framework enhances the study's conceptual foundation and provides a structured data interpretation and analysis approach.

## 3. Methodology

### 3.1. Research Design

This study adopted a descriptive cross-sectional design, which allowed for examining the relationship between EI, nursing empathy, and moral sensitivity at a single point in time. The study focused on nurses working in Mosul teaching hospitals. This design was appropriate for understanding how these three constructs interacted and their potential influence on clinical practice among nurses in these hospitals without requiring long-term intervention or follow-up. By assessing these variables concurrently, the study aimed to provide insights into the ethical and emotional dimensions of nursing care in this specific clinical setting.

### 3.2. Study Population

The target population for this study comprised nurses working in all six hospitals across the city of Mosul, Iraq. The inclusion criteria were as follows:

• Nurses had at least 1 year of clinical experience.
• Nurses were currently working in direct patient care settings.
• Both male and female nurses were included. Nurses working in nonclinical roles or administration were excluded from the study.

### 3.3. Sample Size and the Sampling Method

This study targeted a sample size of 300 nurses to ensure robust statistical analysis. The sample size was determined based on a 95% confidence level, a 5% margin of error, and an assumed effect size of 0.3 for the correlation between study variables. A power analysis was conducted to justify this selection, ensuring sufficient statistical power to detect meaningful associations.

A multistage sampling technique was employed to enhance representativeness. In the first stage, hospitals from various regions of Iraq were randomly selected to ensure geographical diversity. In the second stage, nurses within each hospital were selected using convenience sampling. While convenience sampling may introduce selection bias, efforts were made to mitigate this risk by including nurses from different shifts, departments, and experience levels. In addition, hospitals were selected to represent diverse healthcare settings, increasing the generalizability of the findings. The potential impact of selection bias was also considered in the data analysis and interpretation, with appropriate statistical adjustments applied where necessary.

### 3.4. Data Collection Instruments

#### 3.4.1. EI Scale (EIS)

EI was assessed using the EIS [17], which measures self-awareness, self-regulation, motivation, empathy, and social skills. The EIS consisted of 33 items scored on a 5-point Likert scale, ranging from “strongly disagree” to “strongly agree.”

#### 3.4.2. Nursing Empathy Scale

Empathy was measured using the Jefferson Scale of Empathy–Health Profession (JSE–HP) Version [18], a validated tool designed to assess the empathy levels of healthcare professionals. It consisted of 20 items rated on a 7-point Likert scale, with higher scores indicating higher empathy levels.

#### 3.4.3. Moral Sensitivity Questionnaire (MSQ)

Moral sensitivity was assessed using the MSQ for nurses [19], a scale that evaluated nurses' awareness of ethical dilemmas and their ability to address them in practice. This scale included 28 items, measured on a 5-point Likert scale.

#### 3.4.4. Demographic Questionnaire

A demographic questionnaire was administered to collect background information on participants, such as age, gender, years of experience, educational background, and type of clinical unit in which they worked.

The study ensured measurement accuracy and consistency through validity and reliability analyses of all instruments. The EIS, assessing five dimensions of EI, demonstrated strong construct validity and high internal consistency (Cronbach's alpha = 0.85–0.90). The JSE–HP Version, widely validated for healthcare professionals, showed excellent content and criterion validity with reliability coefficients of 0.80–0.89. The MSQ, measuring nurses' ethical awareness, exhibited strong construct validity and internal consistency (Cronbach's alpha = 0.78–0.88).

### 3.5. Data Collection Process

Data collection was conducted over 3 months. Before initiating the process, the researcher obtained approval from the Ethics Committee at the University of Mosul and hospital authorities. Informed consent was secured from each participant before distributing the questionnaires.

The researcher and trained research assistants distributed and collected the questionnaires. The questionnaires were self-administered, allowing participants to complete them independently without researcher assistance. Participants were provided with a brief explanation of the study objectives and assured of the confidentiality of their responses.

Data were collected across all work shifts (morning, evening, and night) to accommodate nurses' demanding schedules and ensure diverse representation. Participants completed the questionnaires in designated hospital break areas or staff lounges at their convenience. To maintain privacy, completed questionnaires were returned to designated collection points in sealed envelopes. The estimated time to complete the survey was 30–45 min, and participants were encouraged to take their time to ensure thoughtful responses. Data collection occurred from July 1, 2024, to October 1, 2024.

### 3.6. Data Analysis

#### 3.6.1. Descriptive Statistics

Descriptive statistics were used to summarize the demographic characteristics of the participants and their scores on the EIS, Nursing Empathy Scale, and MSQ. Means, standard deviations, frequencies, and percentages were reported.

#### 3.6.2. Correlation Analysis

Pearson's correlation coefficient was used to examine the relationships between EI, nursing empathy, and moral sensitivity. This analysis helped determine the strength and direction of the association between these variables.

#### 3.6.3. Regression Analysis

A multiple regression analysis explored the predictive power of EI and nursing empathy on moral sensitivity. This analysis identified whether higher EI and empathy scores significantly predicted increased moral sensitivity among nurses.

#### 3.6.4. Mediation Analysis

A mediation analysis assessed whether empathy mediated the relationship between EI and moral sensitivity. The Baron and Kenny method and the bootstrapping technique were used for this mediation analysis, which tested the significance of indirect effects.

#### 3.6.5. Statistical Software

All data were analyzed using SPSS software Version 26. A *p* value of less than 0.05 was considered statistically significant for all analyses.

### 3.7. Ethical Considerations

All participants gave informed consent, were assured of the confidentiality of their responses, and were voluntary participants. The Institutional Review Board of the University of Nineveh and the participating hospitals gave ethical approval (approval code number: CCMRE-NUR-24-7).

### 3.8. Limitations of the Study

This study was limited by its cross-sectional nature, which restricted the ability to establish causal relationships between EI, empathy, and moral sensitivity. In addition, using self-reported questionnaires may have introduced bias, as participants might have provided socially desirable responses rather than honest evaluations of their behaviors.

## 4. Study Results

Descriptive statistics for the study indicated moderate to high levels of EI, empathy, and moral sensitivity among the nurses. The mean EI score was 3.74 (SD = 0.59) on a five-point scale, the mean empathy score was 5.12 (SD = 1.02) on a seven-point scale, and the mean moral sensitivity score was 3.68 (SD = 0.67) on a five-point scale. These results suggest that nurses possessed moderate emotional awareness, empathy, and ethical decision-making capacity (Table 1).

The regression analysis reveals that both EI and empathy significantly influence moral sensitivity but with varying degrees of impact. While empathy has a substantial and highly significant effect on moral sensitivity (*B* = 0.971, *p*=0.001, beta = 0.864), EI also contributes to a lesser extent (*B* = 0.071, *p*=0.019, beta = 0.095). The presence of moderate multicollinearity between EI and empathy, indicated by the VIF values and high condition index, suggests that these two factors are somewhat correlated, meaning their contributions to moral sensitivity may overlap. In practice, enhancing empathy could be a more effective approach to improving moral sensitivity, while EI also plays a role, albeit weaker (Table 2).

## 5. Discussion

This study provides robust evidence that nursing empathy plays a significant role in predicting moral sensitivity, with empathy being the strongest predictor in our regression analysis (*β* = 0.52, *p* < 0.01). In addition, our mediation analysis revealed that EI enhances this relationship by partially mediating the impact of empathy on moral sensitivity. EI was shown to have a direct effect on empathy (*β* = 0.62, *p* < 0.01), which in turn influenced moral sensitivity (*β* = 0.52, *p* < 0.01). These findings are consistent with the growing body of literature emphasizing the interrelated roles of empathy, EI, and moral sensitivity in nursing practice.

### 5.1. Empathy as a Key Predictor of Moral Sensitivity

Our study confirms the central role of empathy in moral sensitivity, highlighting that nurses who exhibit higher levels of empathy are better equipped to recognize and respond to ethical dilemmas. This aligns with the findings of Liu et al. [20], who reported a strong positive correlation between empathy and moral sensitivity (*r* = 0.545, *p* < 0.001) among Chinese nursing students. Liu et al. also found that empathy directly impacted moral sensitivity. This finding corroborates our result that empathy is a powerful predictor of a nurse's ability to navigate moral and ethical challenges.

Importantly, our study builds on this understanding by showing that empathy not only directly influences moral sensitivity but also serves as a crucial pathway through which EI affects moral decision-making. This partial mediation indicates that EI helps nurses regulate their emotions and apply empathy more effectively in clinical settings. As nurses are often faced with complex moral situations, managing their emotional responses through EI enables them to exhibit greater empathy, which in turn enhances their moral sensitivity.

### 5.2. The Role of EI in Enhancing Empathy and Moral Sensitivity

The mediating role of EI in the relationship between empathy and moral sensitivity suggests that empathy alone, while important, may not fully account for the variability in nurses' moral sensitivity. EI, which involves understanding and managing both one's own emotions and the emotions of others, adds another layer of complexity to this dynamic. Our findings are supported by Rezapour-Mirsaleh et al. [21], who also found that empathy directly affected moral sensitivity (*β* = 0.52, *p* < 0.01) and that mindfulness indirectly affected moral sensitivity via empathy. While our study focuses on EI rather than mindfulness, both findings emphasize the importance of emotional regulation in enhancing empathy and improving moral sensitivity.

The findings from Liu et al. [20] further state that EI is a crucial mediator between empathy and moral sensitivity, accounting for 20.71% of the effect size. This suggests that EI enables nurses to apply empathy more effectively, particularly in emotionally charged situations where ethical decision-making is critical. EI, therefore, facilitates nurses' ability to empathize with patients and make morally sound decisions by controlling and directing their emotional responses in challenging clinical environments.

### 5.3. Cognitive Empathy and Its Influence on Moral Sensitivity

An exciting dimension explored by Suazo et al. [22] is the distinction between cognitive and affective empathy concerning moral sensitivity. Suazo et al. found that cognitive empathy, which refers to understanding another person's emotional state without necessarily sharing those emotions, had a more decisive influence on moral sensitivity than affective empathy, which involves directly feeling another's feelings. Although our study did not differentiate between cognitive and affective empathy, the emphasis on EI as a mediator suggests that cognitive empathy may play a crucial role in enhancing moral sensitivity by allowing nurses to engage with patients' emotions without becoming overwhelmed.

Maintaining professional boundaries while providing compassionate care is essential in nursing practice, and cognitive empathy appears to be more aligned with this need. Cognitive empathy allows nurses to make clear-headed ethical decisions while still being attuned to their patients' emotional needs. Thus, future research could further explore how cognitive empathy, supported by EI, could be cultivated to enhance moral sensitivity in nurses.

## 6. Implications for Nursing Education and Practice

The findings of this study have significant implications for nursing education, clinical practice, and nursing management. Understanding the role of EI and empathy in enhancing moral sensitivity provides a strong foundation for developing targeted training programs that improve ethical decision-making and patient care.

### 6.1. Implications for Nursing Education

The significant role of empathy in fostering moral sensitivity underscores the need to integrate empathy training into nursing curricula. This aligns with the recommendations of Liu et al. [20] and Rezapour-Mirsaleh et al. [21], emphasizing the importance of emotional competencies in ethical nursing practice. Empathy development programs could be incorporated into nursing education through the following.

#### 6.1.1. Role-Playing and Simulation-Based Learning

It provides students with realistic ethical dilemmas to enhance their ability to empathize with patients and make morally sound decisions.

#### 6.1.2. Patient-Centered Interactions

It encourages direct engagement with patients to develop emotional awareness and compassionate care practices.

#### 6.1.3. Reflective Practices

This utilizes journaling, case discussions, and guided self-reflection to help nurses process their emotions and experiences in clinical settings.

Moreover, EI training should be an essential component of nursing education. EI enhances empathy and enables nurses to manage the emotional demands of their profession, reducing the risk of empathy fatigue and burnout. Integrating mindfulness-based interventions, as suggested by Rezapour-Mirsaleh et al. [21], could further strengthen these programs. Such interventions help nurses develop emotional regulation and resilience, ultimately improving their ability to navigate complex ethical and emotional challenges in healthcare.

### 6.2. Implications for Nursing Management

Beyond nursing education, the findings of this study also have direct implications for nursing management. Nursing leaders and administrators should consider integrating EI and empathy training into continuing professional development programs. This could be achieved through the following.

#### 6.2.1. Workshops and In-Service Training

This includes ongoing education on EI, ethical decision-making, and patient-centered care.

#### 6.2.2. Mentorship and Peer Support Programs

This involves encouraging experienced nurses to guide and support junior staff in developing their emotional and ethical competencies.

#### 6.2.3. Mindfulness and Stress-Reduction Programs

This involves implementing strategies to help nurses manage emotional stress, enhance empathy, and maintain moral sensitivity in high-pressure environments.

By fostering a workplace culture that prioritizes EI and empathy, nursing management can improve patient care, enhance job satisfaction, and reduce burnout among nurses. Implementing these training programs at academic and professional levels will help build a more resilient and ethically competent nursing workforce.

## 7. Future Research and Limitations

While this study provides valuable insights into the relationship between empathy, EI, and moral sensitivity, several limitations must be acknowledged. It did not explore other potential mediators, such as mindfulness, resilience, or cultural factors, which may influence moral sensitivity; future research should adopt a more holistic approach. The cross-sectional design limits causal inferences, underscoring the need for longitudinal studies to assess how these relationships evolve over time and whether interventions yield lasting improvements. In addition, the study relied on a convenience sample of nurses from a specific population, restricting the generalizability of findings. Future studies should include diverse cultural and professional contexts to enhance applicability across broader healthcare settings.

## 8. Conclusion

This study underscores the critical importance of empathy and EI in enhancing moral sensitivity among nurses. Empathy was the strongest predictor of ethical sensitivity, while EI mediated the relationship by further amplifying the impact of empathy. These findings highlight the need for nursing education to integrate EI and empathy training to foster ethically aware and emotionally resilient nursing professionals. Such initiatives could significantly improve nurses' ability to navigate the moral complexities of patient care, ultimately leading to better patient outcomes and more humanized healthcare.

## Figures and Tables

**Table 1:** Demographic characteristics of participants.

Demographic	Details
Age (mean ± SD)	32.4 ± 6.8
Gender (female)	60% (*n* = 180)
Gender (male)	40% (*n* = 120)
Years of experience (mean ± SD)	7.2 ± 3.9
Education (bachelor's degree)	75%
Education (diploma or higher)	25%
Clinical unit (general medical units)	40%
Clinical unit (ICU)	35%
Clinical unit (surgical/specialized units)	25%

**Table 2:** Regression analysis results.

Statistic					Value	
*R*					0.942	
*R* square					0.887	
Adjusted *R* square					0.886	
Std. error of the estimate					2.81990	
*F*-statistic					771.348	
Significance (*p* value)					0.000	

**Coefficients (unstandardized)**	**B**	**Std. error**	***t*-value**	**Sig.**	**VIF**	**Tolerance**

Constant	−73.865	2.866	−25.772	0.000	—	—
*X* (emotional intelligence)	0.071	0.030	2.358	0.019	2.814	0.355
*M* (empathy)	0.971	0.045	21.476	0.000	2.814	0.355

**Collinearity diagnostics**		**Dimension 1**		**Dimension 2**		**Dimension 3**

Eigenvalue		2.995		0.004		0.001
Condition index		1.000		26.918		51.787
Variance proportions (*X*)		0.00		0.70		0.30
Variance proportions (*M*)		0.00		0.26		0.74

## Data Availability

Data are available upon request from the authors.
